# Prevalence of Exclusive Breastfeeding Among US Children

**DOI:** 10.1001/jamanetworkopen.2024.36644

**Published:** 2024-09-27

**Authors:** Guodong Ding, Chaochao Wen, Yan Chen, Angela Vinturache, Yongjun Zhang

**Affiliations:** 1 Department of Pediatrics, Xinhua Hospital, Shanghai Jiao Tong University School of Medicine, Shanghai, China; 2 Department of Obstetrics & Gynecology, University of Alberta, Edmonton, Alberta, Canada

## Abstract

This cross-sectional study analyzes temporal changes in the prevalence of exclusive breastfeeding at 4 and 6 months among US children from 2016 to 2022.

## Introduction

In 2022, the American Academy of Pediatrics and the World Health Organization recommended exclusive breastfeeding for the first 6 months of life, followed by the stepwise introduction of complementary foods up to 2 years of age and beyond.^1^ In contrast, a previous study suggested that solid foods can be safely introduced as early as 4 months of age.^2^ Meanwhile, the current prevalence of 25.4% in 2020^3^ indicates that the Healthy People 2030 goal of achieving a 6-month exclusive breastfeeding rate^4^ is not being met. In this study, we analyzed temporal changes in the prevalence of exclusive breastfeeding at 4 and 6 months among US children from 2016 to 2022.

## Methods

This cross-sectional study used deidentified, publicly available data and was therefore exempt from institutional review board approval in accordance with the Common Rule. Written informed consent for data collection was obtained from children’s parents or other primary caregivers. We followed the STROBE reporting guideline.

The National Survey of Children’s Health (NSCH) is a US survey for monitoring the health and well-being of noninstitutionalized children aged 0 to 17 years.^5^ Data are collected annually from parents or other primary caregivers through mail- or web-based questionnaires. We used NSCH data from 2016 to 2022 (which were released on April 24, 2024) to evaluate temporal changes in the prevalence of exclusive breastfeeding at 4 and 6 months among children aged 0 to 1 year. Self-reported race and ethnicity data were collected to assess disparities among different racial and ethnic groups.

Inclusion criteria for children were as follows: (1) be at least 4 or 6 months old, (2) did not stop breastfeeding before 4 or 6 months of age, and (3) were not introduced to formula or food other than breast milk (even water) until at least 4 or 6 months of age. Weighted data estimated the prevalence of exclusive breastfeeding at 4 and 6 months of age. Temporal changes in slopes (up to one joinpoint) were assessed using Joinpoint, version 5.0.2 (National Cancer Institute) by calculating the average annual percent change and 95% CI, both overall and by sociodemographic subgroup. Two-tailed *P* < .05 indicated statistical significance.

## Results

Among 21 860 children, 658 (3.0%) with missing or unreliable breastfeeding data were excluded. We also excluded 2941 children who were younger than 4 months and 4409 who were younger than 6 months, resulting in 18 261 (51.7% boys) and 16 793 (51.9% boys) participants for analysis of exclusive breastfeeding at 4 and 6 months of age, respectively. The overall prevalence of exclusive breastfeeding at 4 months was 33.5% in 2016 and 37.5% in 2022 (Figure). The prevalence of exclusive breastfeeding at 6 months of age increased from 20.5% (95% CI, 17.6%-23.4%) in 2016 to 26.1% (95% CI, 23.4%-28.8%) in 2022, with a 3.0% (95% CI, 0.4%-5.7%) relative increase per year (*P* = .03 for trend) (Table).

**Figure.**
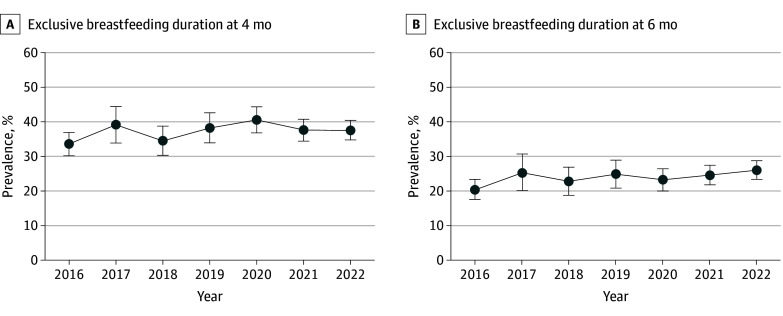
Prevalence of Exclusive Breastfeeding at 4 and 6 Months Among US Children, 2016 to 2022 Data were obtained from the National Survey of Children’s Health and were weighted to be nationally representative. Error bars indicate 95% CIs. A, For exclusive breastfeeding at 4 months, the average annual percent change (95% CI) was 1.5 (−1.3 to 4.4) (*P* = .22 for trend). B, For exclusive breastfeeding at 6 months, the average annual percent change (95% CI) was 3.0 (0.4 to 5.7) (*P* = .03 for trend).

**Table.** Prevalence of Exclusive Breastfeeding Duration at 6 Months Among US Children Aged 6 Months to 1 Year, 2016 to 2022^a^

Exclusive breastfeeding at 6 mo^b^	Year							Overall	Mean APC (95% CI)	*P* value for trend
	2016	2017	2018	2019	2020	2021	2022			
Total	838 (20.5 [17.6 to 23.4])	343 (25.4 [20.0 to 30.7])	430 (22.9 [18.8 to 27.0])	410 (24.9 [20.9 to 28.9])	644 (23.3 [20.1 to 26.5])	999 (24.7 [21.8 to 27.5])	1000 (26.1 [23.4 to 28.8])	4664 (24.0 [22.5 to 25.4])	3.0 (0.4 to 5.7)	.03
Sex of child^c^										
Boys	428 (19.0 [15.6 to 22.3])	166 (26.5 [18.2 to 34.9])	220 (21.9 [16.2 to 27.6])	213 (24.4 [19.5 to 29.3])	336 (23.9 [19.3 to 28.5])	488 (21.5 [18.0 to 25.0])	502 (27.8 [24.0 to 31.6])	2353 (23.6 [21.5 to 25.6])	4.5 (−0.7 to 9.9)	.08
Girls	410 (22.2 [17.5 to 26.9])	177 (24.1 [17.6 to 30.6])	210 (24.0 [18.2 to 29.9])	197 (25.4 [19.1 to 31.8])	308 (22.6 [18.3 to 27.0])	511 (28.0 [23.5 to 32.5])	498 (24.3 [20.5 to 28.2])	2311 (24.4 [22.4 to 26.4])	2.0 (−1.9 to 6.0)	.26
Race and ethnicity of child^c^										
Hispanic	78 (13.0 [8.1 to 17.8])	31 (24.2 [7.5 to 41.0])	56 (25.1 [12.6 to 37.7])	40 (19.2 [9.5 to 29.0])	68 (17.1 [8.9 to 25.2])	121 (18.4 [12.4 to 24.5])	148 (21.2 [16.1 to 26.4])	542 (19.9 [16.0 to 23.7])	4.6 (−3.9 to 13.9)	.23
Non-Hispanic Black	24 (13.2 [1.5 to 25.0])	9 (34.4 [12.1 to 56.7])	19 (12.3 [5.0 to 19.6])	13 (12.7 [3.2 to 22.1])	29 (25.9 [13.3 to 38.5])	29 (18.9 [6.8 to 31.0])	31 (20.4 [8.9 to 31.9])	154 (19.1 [14.1 to 24.1])	2.5 (−17.3 to 27.2)	.78
Non-Hispanic White	650 (25.9 [22.4 to 29.4])	266 (26.4 [21.5 to 31.4])	318 (24.7 [20.7 to 28.7])	310 (30.4 [25.6 to 35.2])	457 (26.0 [22.5 to 29.5])	722 (28.8 [25.4 to 32.2])	699 (30.2 [26.7 to 33.7])	3422 (27.4 [25.9 to 28.9])	2.5 (−0.5 to 5.7)	.09
Other^d^	86 (21.1 [13.3 to 28.9])	37 (18.4 [7.2 to 29.7])	37 (21.1 [9.9 to 32.4])	47 (25.5 [14.6 to 36.4])	90 (20.5 [13.2 to 27.8])	127 (24.0 [15.9 to 32.0])	122 (25.5 [18.7 to 32.2])	546 (22.1 [18.4 to 25.8])	3.5 (−0.6 to 7.8)	.08
Family income, % FPL^e^										
<100	52 (9.7 [5.4 to 14.0])	29 (24.4 [5.9 to 42.9])	35 (18.7 [8.7 to 28.8])	33 (13.4 [7.0 to 19.7])	61 (15.3 [8.4 to 22.2])	71 (17.9 [10.4 to 25.4])	89 (20.6 [14.4 to 26.8])	370 (16.9 [13.2 to 20.6])	9.1 (−1.7 to 21.1)	.09
100-199	115 (21.1 [12.4 to 29.9])	40 (17.3 [8.1 to 26.5])	70 (20.9 [13.2 to 28.5])	70 (21.8 [15.2 to 28.3])	98 (22.0 [13.7 to 30.3])	151 (19.7 [14.9 to 24.5])	132 (21.9 [16.9 to 26.9])	676 (20.6 [17.7 to 23.5])	0.8 (−2.8 to 4.5)	.60
200-399	285 (25.1 [20.0 to 30.3])	116 (27.1 [19.4 to 34.8])	138 (26.9 [20.6 to 33.2])	138 (30.5 [23.0 to 38.0])	226 (30.2 [24.3 to 36.2])	317 (25.6 [20.6 to 30.6])	315 (28.2 [23.3 to 33.1])	1535 (27.6 [25.2 to 30.0])	1.3 (−2.3 to 4.9)	.41
≥400	386 (24.2 [20.1 to 28.4])	158 (29.2 [22.0 to 36.3])	187 (24.0 [18.1 to 30.0])	169 (29.5 [22.5 to 36.5])	259 (23.5 [18.9 to 28.1])	460 (30.1 [25.7 to 34.6])	464 (29.4 [25.1 to 33.8])	2083 (27.3 [25.1 to 29.4])	2.9 (−1.7 to 7.6)	.17
Highest education of parent or primary caregiver										
Less than college^f^	177 (12.2 [8.9 to 15.5])	83 (23.8 [13.1 to 34.4])	110 (15.6 [10.4 to 20.8])	101 (14.1 [9.9 to 18.3])	171 (18.9 [13.6 to 24.3])	217 (16.4 [12.4 to 20.3])	245 (20.9 [16.8 to 25.0])	1104 (17.4 [15.1 to 19.7])	6.5 (−1.8 to 15.6)	.10
College or higher	660 (28.3 [23.9 to 32.6])	260 (26.6 [21.4 to 31.7])	320 (29.8 [24.0 to 35.6])	309 (32.9 [27.2 to 38.5])	473 (27.2 [23.5 to 31.0])	782 (30.7 [26.9 to 34.5])	755 (29.9 [26.4 to 33.4])	3559 (29.3 [27.6 to 31.1])	1.1 (−2.0 to 4.4)	.40
Maternal age at delivery, y										
≤30	433 (20.6 [17.0 to 24.2])	157 (16.6 [11.5 to 21.8])	197 (20.0 [14.0 to 26.0])	159 (18.8 [14.7 to 22.8])	278 (23.6 [19.2 to 28.1])	401 (22.0 [18.1 to 25.9])	368 (24.7 [20.6 to 28.9])	1993 (20.8 [19.0 to 22.5])	3.7 (−0.6 to 8.1)	.08
>30	398 (22.0 [17.2 to 26.9])	181 (33.1 [24.5 to 41.7])	232 (25.8 [20.2 to 31.4])	248 (30.1 [23.9 to 36.3])	360 (23.2 [18.6 to 27.8])	588 (26.3 [22.3 to 30.4])	613 (26.9 [23.3 to 30.5])	2620 (26.9 [24.7 to 29.0])	0.6 (−5.3 to 6.8)	.82
Smoker in household^g^										
Yes	56 (10.8 [5.2 to 16.4])	28 (13.6 [6.9 to 20.3])	24 (15.6 [7.3 to 23.9])	33 (17.3 [9.3 to 25.4])	44 (7.9 [4.5 to 11.3])	38 (10.8 [5.2 to 16.4])	43 (14.1 [7.9 to 20.4])	266 (12.7 [10.3 to 15.2])	−1.4 (−15.4 to 14.9)	.82
No	778 (22.4 [19.1 to 25.7])	307 (26.6 [20.6 to 32.7])	401 (24.4 [19.9 to 29.0])	369 (26.3 [21.8 to 30.7])	591 (25.5 [21.9 to 29.1])	942 (25.9 [22.8 to 29.1])	941 (27.6 [24.6 to 30.5])	4329 (25.6 [24.0 to 27.2])	2.7 (0.7 to 4.7)	.02

Abbreviations: APC, annual percent change; FPL, federal poverty level.

^a^ Unless indicated otherwise, data are presented as No. (% [95% CI]). Data were obtained from the National Survey of Children’s Health. All estimates were weighted except for sample sizes. For certain subgroups, numbers may not sum to the total number owing to missing data.

^b^ Exclusive breastfeeding was defined as receiving only breast milk (no solids, no water, and no other liquids).

^c^ Missing data were imputed by the US Census Bureau using hot deck imputation.

^d^ Includes American Indian or Alaska Native, Asian, Native Hawaiian or Other Pacific Islander, and multiple races or ethnicities.

^e^ Missing data were imputed by the US Census Bureau using sequential regression imputation methods.

^f^ Includes less than high school, high school, and some college or technical school.

^g^ Defined according to responses to the question “Does anyone living in your household use cigarettes, cigars, or pipe tobacco?”

No association was found between sociodemographic factors and exclusive breastfeeding practices (Table). Exclusive breastfeeding at 6 months of age was more predominant in nonsmoking households (Table).

## Discussion

The prevalence of exclusive breastfeeding among US children at 4 months of age increased only slightly throughout the study period, with fewer than half meeting the aforementioned recommendations for exclusive breastfeeding. Although the overall prevalence of exclusive breastfeeding at 6 months also increased during the study period, it remained below the Healthy People 2030 goal of 42.4%.^4^ These findings suggest that three-quarters of US children are not receiving the maximum health benefits associated with exclusive breastfeeding.

The nationally representative study population and quality of data render our findings reliable and generalizable. However, our study is limited in that the data do not allow for causal inferences regarding factors that may affect exclusive breastfeeding practices. Further investigation is needed to identify and understand the causes of early abandonment of breastfeeding. Such research will help support women and their families in continuing to provide infants with optimal nutrition for proper development.

## Notes

Supplement.
Data Sharing Statement

## References

[1] Meek JY, Noble L; Section on Breastfeeding. Policy statement: breastfeeding and the use of human milk. Pediatrics. 2022;150(1):e2022057988. doi:10.1542/peds.2022-057988 35921640

[2] Fewtrell M, Wilson DC, Booth I, Lucas A. Six months of exclusive breast feeding: how good is the evidence? BMJ. 2010;342:c5955. doi:10.1136/bmj.c5955 21233152

[3] Results: breastfeeding rates. Centers for Disease Control and Prevention. August 1, 2023. Accessed May 1, 2024. https://www.cdc.gov/breastfeeding/data/nis_data/results.html

[4] Healthy People 2030: building a healthier future for all. US Department of Health and Human Services, Office of Disease Prevention and Health Promotion. Accessed January 2, 2024. https://health.gov/healthypeople/objectives-and-data/browse-objectives/nutrition-and-healthy-eating

[5] NSCH datasets. Revised September 28, 2023. US Census Bureau. Accessed September 28, 2023. https://www.census.gov/programs-surveys/nsch/data/datasets.html

