# Supplementary material for: Prevalence of Exclusive Breastfeeding Among US Children
Source: JAMA Netw Open. 2024 Sep 27;7(9):e2436644. doi: 10.1001/jamanetworkopen.2024.36644 (PMC11437381; doi:10.1001/jamanetworkopen.2024.36644)
Supplement: Supplement. — Data Sharing Statement [file jamanetwopen-e2436644-s001.pdf]

Data Sharing Statement

Ding. Prevalence of Exclusive Breastfeeding Among US Children. *JAMA Netw Open*.
Published October 01, 2024. doi:10.1001/jamanetworkopen.2024.36644

Data

Data available: Yes
Data types: Deidentified participant data
How to access data: Data sharing will be available from YZ upon a reasonable request. Electronic address: zhangyongjun@sjtu.edu.cn.
When available: beginning date: 10-01-2024, end date: 10-01-2025

Supporting Documents

Document types: Statistical/analytic code
How to access documents: Data sharing will be available from YZ upon a reasonable request. Electronic address: zhangyongjun@sjtu.edu.cn.
When available: beginning date: 10-01-2024, end date: 10-01-2025

Additional Information

Who can access the data: Data sharing will be available from YZ upon a reasonable request. Electronic address: zhangyongjun@sjtu.edu.cn.
Types of analyses: Data sharing will be available from YZ upon a reasonable request. Electronic address: zhangyongjun@sjtu.edu.cn.
Mechanisms of data availability: Data sharing will be available from YZ upon a reasonable request. Electronic address: zhangyongjun@sjtu.edu.cn.
Any additional restrictions: Data sharing will be available from YZ upon a reasonable request. Electronic address: zhangyongjun@sjtu.edu.cn.
